# Effects of Glucosamine and Chondroitin Sulfate on Cartilage Metabolism in OA: Outlook on Other Nutrient Partners Especially Omega-3 Fatty Acids

**DOI:** 10.1155/2011/969012

**Published:** 2011-08-02

**Authors:** Jörg Jerosch

**Affiliations:** Department of Orthopedics, Trauma Surgery and Sports Medicine, Johanna-Etienne Hospital, 41462 Neuss, Germany

## Abstract

Osteoarthritis (OA) is a degenerative joint disease that is characterized by increasing loss of cartilage, remodeling of the periarticular bone, and inflammation of the synovial membrane. Besides the common OA therapy with nonsteroidal anti-inflammatory drugs (NSAIDs), the treatment with chondroprotectives, such as glucosamine sulfate, chondroitin sulfate, hyaluronic acid, collagen hydrolysate, or nutrients, such as antioxidants and omega-3 fatty acids is a promising therapeutic approach. Numerous clinical studies have demonstrated that the targeted administration of selected micronutrients leads to a more effective reduction of OA symptoms, with less adverse events. Their chondroprotective action can be explained by a dual mechanism: (1) as basic components of cartilage and synovial fluid, they stimulate the anabolic process of the cartilage metabolism; (2) their anti-inflammatory action can delay many inflammation-induced catabolic processes in the cartilage. These two mechanisms are able to slow the progression of cartilage destruction and may help to regenerate the joint structure, leading to reduced pain and increased mobility of the affected joint.

## 1. Introduction

Osteoarthritis (OA), the most common type of arthritis, is characterized by gradual wear and loss of cartilage in the joints resulting in friction between the bones, which leads to pain and swelling. It was long thought that only the cartilage is affected. However, it is now known that the underlying bone, as well as the synovium, also undergoes changes [1–3]. The periarticular bone reacts with osteophyte formation which causes additional restriction in joint movement. It can occur in any joint, but predominates in weight-bearing joints, such as the knee and hip. In Germany, the prevalence of diagnosed osteoarthritis (all age groups combined) in at least one joint is 27%, and more than 50% of the population over 60 suffer from OA in at least one joint [4]. In the United States of America, OA is responsible for total joint replacement in half a million Americans each year [5], indicating that OA is not only a burden to the patients, but also a financial burden on society.

Common OA therapy focuses mainly on the treatment of symptoms, such as pain reduction, but does not treat the cause. However, the main goal of OA therapy should be to delay cartilage degeneration and even help to regenerate the cartilage structure. One approach in this direction is the treatment with chondroprotectives, differentiated in symptomatic slow-acting drugs in OA (SYSADOA) or structure-modifying OA drugs (SMOAD).

This paper will focus on the ability of such chondroprotectives to retard the degenerative process of cartilage destruction and will discuss the evidence of symptomatic and structure-modifying effects of this nutritional approach. Furthermore, the role of inflammation and especially obesity in the process of osteoarthritis and how this process could be addressed will be discussed.

## 2. Common Risk Factors for the Development of Osteoarthritis

There are still questions concerning the *causal factors of OA.* The nature of the initiating event is often unknown, although many processes involved in the progression of OA are known. Due to disruption of the cartilage collagen matrix, the water content of the cartilage increases. Together with the progressive loss of proteoglycans, the elasticity of the cartilage diminishes. This is followed by a progressive loss of cartilage and the formation of osteophytes and calcium deposits. Osteophytes further limit flexibility of the joint. OA progression is associated with synovial inflammation, joint swelling, stiffness and pain, leading to progressive functional impairment [5, 6].

There are several known *risk factors*. One of the primary risk factor for OA is *age* [3, 7]. During aging, the articular cartilage softens. The ability to remodel and repair the cartilage extracellular matrix (ECM) decreases with age [8]. Furthermore, changes are due to the structural organization of the ECM [9, 10]. During aging, cross-linking of collagen fibers is enhanced which results in increased cartilage stiffness [11]. Aging also leads to reduced muscle mass and strength, which in turn reduces joint stability and leads to misalignment. This can cause abnormal mechanical stress on the joint and thus cartilage degeneration [12].

Another commonly accepted risk factor is *overweight and obesity*. A recent meta-analysis addressed the incidence of comorbidity related to overweight and obesity. It was able to show that overweight and obesity lead to a significantly higher OA risk [13]. The mechanisms by which obesity contribute to OA development are described below.

A number of studies demonstrated that there are strong *genetic determinants* for OA (for review see [14, 15]). A classic twin study in which twins were radiologically screened for OA, showed a clear genetic influence on hand and knee OA in women. Hence the genetic influence was calculated to be 39–65% [16]. Several genetic abnormalities have been identified that are responsible for the onset and progression of OA. These gene variations result in defects or variability of cartilage and ECM composition and metabolism [14, 17, 18].

Patients with *developmental dysplasia* of joints, such as hip dysplasia, develop OA much earlier than normal individuals. *Misalignment* leads to a reduced contact area within the joint resulting in locally elevated pressure on the cartilage [19]. This is related to the progression and onset of OA [20, 21]. *Injuries* involving the joint surface, injured ligaments, or meniscectomy, are also associated with the development of OA. Injuries can often cause joint movement beyond the physiological range which leads to *uneven load distribution* in the joint.

Despite the difference in the primary causes of OA, they all lead to similar clinical symptoms, cartilage destruction, bone remodeling, osteophyte formation, inflammation of the synovial membrane, pain, and immobility.

## 3. Regeneration of the Cartilage Structure

### 3.1. Basic Structure and Turnover of Normal Joint Cartilage

To understand the structure-modifying effect of different nutrients and how they can support the process of cartilage regeneration, it is important to know the composition of cartilage and the metabolic mechanisms involved in normal turnover.

Cartilage is classified into three different types, based on the collagen type used and the relative amount of the main components, that is, elastic cartilage, hyaline cartilage, and fibrocartilage. Unlike other tissue it is not innervated and does not contain blood vessels or lymphatic structures. There are only a small number of chondrocytes within the cartilage and they only account for 1–5% of the cartilage volume. The chondrocytes are responsible for maintaining the composition and organization of the matrix. They produce this extracellular matrix composed of collagen and elastin fibers, as well as proteoglycans. 

*Hyaline cartilage*, found in joints, is characterized by its high elasticity and pressure resistance. In contrast to bone and muscle, it does not increase its tissue mass postnatally due to mechanical stimulation. The morphology of cartilage seems to be strongly related to genetic factors [22]. It is composed of four different zones: the superficial tangential zone, the middle or transitional zone, the deep or radial zone, and the calcified cartilage zone [23, 24]. The collagen network of the joint cartilage consists mainly of type II collagen fibrils. Collagen fibers are important for the response to tensile forces within the joint.

**Figure 1:**
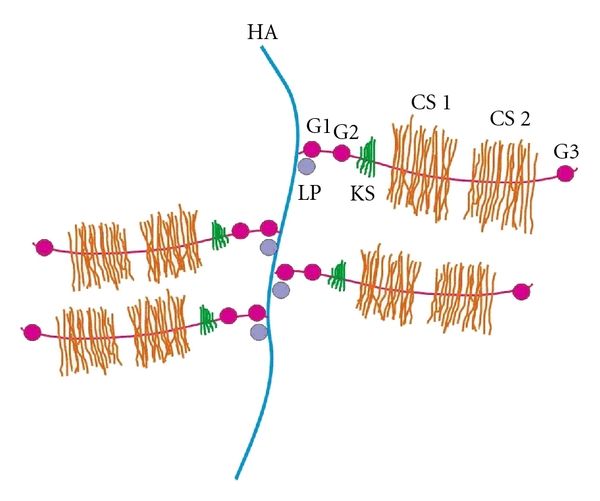
Schematic representation of the aggrecan structure (HA: hyaluronic acid, CS 1, CS 2: chondroitin sulfate domains 1 and 2; KS: keratan sulfate; G1, G2, G3: globular domains; LP: link protein).

*Proteoglycans* are intertwined with the collagen network. Due to the net negative charge of the proteoglycans, a large amount of water is enclosed in the cartilage. The water content is important for the resilience and elasticity of the tissue, as well as for lubrication of the joint system. The proteoglycans of the articular cartilage are large supramolecular complexes, composed of a central hyaluronic acid (HA) filament, to which aggrecan molecules composed of chondroitin sulfate and keratan sulfate are attached by a link protein in a brush-like configuration (see Figure 1). The amino sugar glucosamine is a necessary component for the synthesis of many of these proteoglycans, which include hyaluronic acid, heparan sulfate, and keratan sulfate. The production of glucosamine is one of the rate-limiting steps in proteoglycan production.

The ability of the *articular cartilage* to regenerate or adapt to mechanical changes is very limited. It has been postulated that this inability to adapt to mechanical changes is related to its inability to repair after mechanical or other damage [25]. One reason is the avascular nature of this tissue, which makes it difficult to move progenitor cells to lesion sites. In *in vivo* models of rabbits and goats, it has been shown that lesions smaller than 3 mm in diameter can heal (chondral or subchondral zone) while defects larger than 6 mm in diameter rarely if ever heal and lead to progressive degeneration (for review see [26]). 

Due to the lack of blood vessels, the chondrocytes within the cartilage receive nutrients only by diffusion from the surrounding tissue. Therefore, a large amount of basic components should be available in that tissue.

The viscous *synovial fluid* is composed of hyaluronic acid (hyaluronan), lubricin (a large, water-soluble glycoprotein), glucose, and water. Hyaluronan is synthesized by the synovial membrane and released into the joint cavity.

### 3.2. Chondroprotectives

As shown above, glucosamine, hyaluronic acid, and chondroitin sulfate are important basic natural components of cartilage and synovial fluid. They are naturally formed by the body, but can also be provided in the diet. 

Supplementation of such basic components may be beneficial, especially when there is a disturbed balance between catabolic and anabolic processes, such as in osteoarthritis. During OA progression, the chondrocytes are no longer able to fully compensate for the loss of collagen type II fibers and proteoglycans, even at increased synthesis rates [24].

It has been shown in many *in vitro* and *in vivo* trials and in numerous clinical studies that these SMOAD can modify, stabilize, retard, or even reverse the pathology of OA.

#### 3.2.1. Glucosamine Salts

Glucosamine or 2-amino-2-deoxy-D-glucose (C_6_H_13_NO_5_) is an amino monosaccharide. It is synthesized from glucose in almost every human tissue and is most abundant in connective tissue and cartilage. Glucosamine can be extracted from chitin, found primarily in the exoskeleton of crustaceans (crabs, prawns, and lobsters), as well as in the cell membranes of mushrooms. It is an important precursor of the glycoprotein and glycosaminoglycan (GAG) synthesis. Within cartilage, it is most important for the formation of hyaluronic acid, chondroitin sulfate as well as keratan sulfate, which are—aside from the collagen fibers—the most important components of the extracellular matrix of the articular cartilage and the synovial fluid (for review see [6, 44, 45]). Glucosamine production is the rate-limiting step in GAG synthesis, and glucosamine supplementation may overcome this bottleneck.

Due to its basic role in cartilage and synovial fluid synthesis, glucosamine—administered as glucosamine sulfate (GlcN·S) or hydrochloride (GlcN·HCl)—has been tested in numerous clinical OA trials and the effects have been summarized in reviews and meta-analyses [6, 33, 34, 37, 38, 44–50].

A recent comprehensive review published in 2010 [51], summarized, on the basis of peer-reviewed publications, the currently available chemical and pharmacokinetic data of GlcN salts, and their role in the treatment of clinical OA. An important aspect of GlcN is the structure of various oral GlcN compounds: regardless of the nature of the salt, GlcN·HCl or GlcN·S, the organic component glucosamine is structurally identical. GlcN·HCl dissociates completely in the stomach to GlcN and HCl, and GlcN·S dissociates to GlcN, HCl, sodium sulfate, and sulfuric acid. Investigators have claimed in favor of the GlcN sulfate salt that the sulfate anion would stimulate the chondroitin sulfate synthesis, however, to achieve this serum concentrations of 50 times the serum sulfate concentration would be necessary [51].

In horse studies (see e.g., [52]) *C*_max_ was about 10 *μ*M at 2 h, and here also, the sulfate and chloride salts of GlcN were essentially identical. In human volunteers *C*_max_ was determined to be between 1 and 4 hours after ingestion of a dose of 20 mg GlcN·S per kg body weight (for a typical adult with a body weight of 75 kg, this corresponds to a daily dose of 1500 mg). In four pharmacokinetic studies in humans, maximum serum levels were between 9 and 11 *μ*m, and in one group of OA patients, mean *C*_max_ was 7 *μ*M. Laverty et al. [52] were the first to demonstrate that free GlcN can be detected in synovial fluid after administration (cited in [53]). They found that the synovial fluid concentrations of GlcN remained elevated in most animals even at 12 h after administration. This is in contrast to the nearly complete clearance of GlcN in serum 6 hours after dosing.

***In Vitro* Studies**

*In vitro* studies on isolated chondrocytes, or cartilage explants from healthy or OA patients, provide much evidence for the proposed mechanisms regarding how glucosamine supports joint health. It has been shown that glucosamine enhances the production of cartilage matrix components in chondrocyte culture, such as aggrecan and collagen type II [54, 55]. Glucosamine increases hyaluronic acid production in synovium explants [56]. Further experiments have shown that glucosamine prevents collagen degeneration in chondrocytes by inhibiting lipoxidation reactions and protein oxidation [57]. MMPs (matrix metalloproteinases) and aggrecanases are the predominant cleavage enzymes in the cartilage. These enzymes are responsible for cleavage preferentially in the interglobular domain of the aggrecan molecule, which leads to loss of aggrecan function [24]. Glucosamine is able to inhibit the MMP synthesis, and further proteoglycan degeneration is therefore prevented [58, 59]. Glucosamine also inhibits aggrecanase by suppression of glycosylphosphatidylinositol-linked proteins [60]. Inflammatory processes, which are also responsible for degeneration of the cartilage, are inhibited by glucosamine. These mechanisms will be explained in Section 4.

**Selected Clinical Trials**

The summarized data of major clinical trials (RCTs) between 2001 and 2007 with form of glucosamine used, active reference agents, patient characteristics, outcome measure, and results are listed in Table 1.

The positive effects of glucosamine on the progression of knee OA was not shown in patients suffering from hip OA. In a recent clinical trial, GlcN·S (1500 mg/day) was not able to show superiority over placebo [61], even when a subgroup analysis of the available data was made [62]. The reason why GlcN·S is effective for knee OA, but not for hip OA, is unclear.

Furthermore, it is not understood why many trials stated that there was a significant superiority of GlcN·S over placebo or NSAIDs (e.g., Qiu et al. [63]), whereas others did not. Other trials failed to achieve significance due to a high placebo effect. The heterogeneity of the subjects was also a possible reason, as well as bias due to industry funding. The opinions on this differ and have recently caused much debate [35, 64, 65].

**Selected Reviews and Meta-Analyses**

The quality of evidence was recently evaluated by comparing data from clinical studies, meta-analyses, and reviews (published between 1950 and 2007) on the effect of SYSADOA, including glucosamine sulfate [33]. Using a specialized rating method (GRADE), 5 meta-analyses and one comprehensive review were identified which were included in the evaluation of glucosamine sulfate. Based on this data, it was concluded that glucosamine sulfate, among others, has “demonstrated pain reduction and physical function improvement with very low toxicity, with moderate to high quality evidence” [33]. The results of the Cochrane review by Towheed et al. [34] were included in their evaluation.

The summarized data of selected systematic reviews/meta-analyses, published between 2005 and 2008 with their conclusions are listed in Table 2.

In most trials, dosages of 1500 mg/day were used; the dose was as safe as placebo and was tolerated better than NSAIDs.

From the clinical trials, it can be concluded that long-term treatment with glucosamine:

- reduces pain,
- improves function/mobility of the joint,
- reduces OA progression,
- reduces risk of total joint replacement.

The European League Against Rheumatism (EULAR) came to similar conclusions and rated GlcN·S in their guidelines for knee OA with the highest level of evidence, 1A, and recommended its use with an A [66].

The results of all these studies demonstrate that glucosamine has many favorable effects on cartilage. First, it has shown an anabolic stimulating effect on cartilage synthesis. Furthermore, it inhibits by means of several anti-inflammatory and antioxidant mechanisms, the catabolic cartilage degenerating reactions observed in OA (see Section 4). This can delay cartilage degeneration in OA which leads to a reduction in pain and swelling as well as to increased mobility of the affected joint.

#### 3.2.2. Chondroitin Sulfate

Chondroitin sulfate (CS) is one of the natural glycosaminglycans (GAG) composed of the alternating sugars D-glucuronic acid (GlcA) and N-acetyl-D-galactosamine (GalNAc). It is an important component of the extracellular matrix (ECM). CS is the most frequent GAG in the aggrecan molecule of the cartilage. Due to the negative charge of CS, it is responsible for the water retention of the cartilage, which is important for pressure resistance. It can be extracted from the cartilaginous tissue of cows, pigs, birds, and fish (sharks) and is ingested in the diet.

In the European League Against Rheumatism (EULAR) recommendation concerning knee OA, they gave CS both the highest evidence grade and the highest recommendation strength, 1A and A, respectively [66]. CS is one of the SYSADOA. The first effects of SYSADOA treatments, other than analgesics and NSAIDs, become noticeable after 2 to 3 weeks of regular intake and has a prolonged effect that remains for up to several months. CS influences the symptoms of OA such as pain and inflammation, but also acts as a structure-modifying drug in OA (SMOAD). It may retard OA progression and could modify the course of OA (for review see [39]; details from this systematic review on the clinical use of oral CS in OA is provided in Table 3).

The ability of CS to slow down the development of OA has been demonstrated in several clinical trials [43, 67, 68]. These results were confirmed in a recent long-term study (see also Table 3 for trial data; [42]). With this study, the authors were able to confirm the results of a study performed previously (see Table 3; [43]).

The positive impact of CS on OA was also confirmed by meta-analyses, which all showed a significant favorable effect of CS over placebo [33, 40, 41]. Another comprehensive review of CS was written by the Natural Standard Monograph team. These authors listed 39 clinical studies or meta-analyses in which CS was used to treat OA. Most of these studies came to the conclusion that CS has a significant positive effect on OA patients [69].

One of the studies without a significant effect was the GAIT study [28] (see Table 1 for further details). In that study, intake of CS resulted in only a 5.3% higher responder rate than placebo, which was not statistically significant. However, treatment with CS led to a statistically significant improvement in knee joint swelling [28]. The statistical nonsuperiority of CS in pain reduction can probably be explained by the unexpectedly high placebo effect in this study (61% responder). All of the studies and meta-analyses [37, 40, 41, 70] gave CS an excellent safety profile, therefore there are no safety concerns for long-term use [71].

Similar to the GAIT study, many clinical studies tested chondroitin sulfate together with glucosamine [6, 47, 72–74]. The results suggest that both components may enhance each other's efficacy. This synergistic effect was also proposed by various *in vivo* and *in vitro* studies [55, 75–78].

CS increases the hyaluronan production by human synovial cells, which has a beneficial effect on maintaining viscosity in the synovial fluid [79]. It has been shown that CS stimulates the chondrocyte metabolism, leading to the synthesis of collagen and proteoglycan, the basic components of new cartilage. Furthermore, CS inhibits the enzymes leukocyte elastase and hyaluronidase, which are found in high concentration in the synovial fluid of patients with rheumatic diseases. CS also increases the production of hyaluronic acid by synovial cells, which subsequently improves the viscosity and the synovial fluid levels. In general, CS inhibits cartilage destruction processes and stimulates the anabolic processes involved in new cartilage formation (for review see [6, 69]). In addition, CS, when added to chondrocyte cultures, produces a dose-dependent increase in cell proliferation. 

Several mechanisms are discussed which lead to the positive impact of CS on OA patients. Pharmacokinetic studies were able to show that orally ingested chondroitin sulfate is absorbed as a high molecular mass polysaccharide and can be detected in plasma, together with derivatives, resulting from partial depolymerization and/or desulfation [80]. A pharmacokinetic study (1990) in rats and dogs [81] tested the distribution of tritiated CS orally and intramuscularly. More than 70% of the orally administered radioactivity was absorbed. Independently of the route of administration, radioactivity was mainly excreted through the urine. Plasma levels showed a rapid increase after oral administration, followed by a large plateau with a maximum after 14 or 28 hours in rats and dogs, respectively.

In the years after the publication of the GAIT study, using a combination of GlcN·HCl and CS, new pharmacokinetic data in humans, for both chondroprotectives became available. Jackson et al. [82] tried to assess the pharmacokinetic behavior of oral GlcN and CSeither separately or combined. First they found that the basal levels of GlcN in plasma were at any time below the detection limit, while with CS plasma levels were approximately. 20 *μ*g/mL and did not show any circadian variation. In a second trial phase, they examined the pharmacokinetics of 1500 mg of GlcN·HCl, 1200 mg CS, or a combination of both substances. In a third phase, they selected a group of patients with symptomatic knee OA (as part of GAIT) who had already received 1500 mg GlcN·HCl, 1200 mg CS, or a combination of both for more than 3 months every day. The main finding was that none of the experimental procedures led to alterations in the endogenous plasma CS concentration. The basal GlcN levels in plasma which had not been detectable before increased, but with combined administration together with CS were significantly reduced.

The authors concluded that the clinical improvement of OA symptoms which was obvious in the numerous clinical trials (also for a subgroup of the GAIT patient population, [28]) is not caused by a synergistic effect of both agents during intestinal absorption, but that there may be indirect effects of these two agents on joint health. They hypothesize that the favorable clinical effects of both compounds may result from “changes in cellular activities in the gut lining or in the liver, where concentrations of ingested CS, or its breakdown products, could be substantially elevated following oral ingestion” [82].

In summary, all the information from these *in vitro *and *in vivo *studies, the clinical trials, as well as meta-analyses lead to the conclusion that there is sufficient data to support the use of oral CS in OA. The findings show that CS reduces pain, improves function/mobility of the joint, and reduces the progression of OA by its structure-modifying effects.

#### 3.2.3. Other Compounds

In addition to the combination GlcN·S + CS, other related substances, for example, *hyaluronic acid* (HA, hyaluronan) and collagen hydrolysate, have been used in OA patients.

Regarding therapeutical use of HA, the backbone of a proteoglycan aggregate within the ECM, not all clinical trials reported the same positive result. It seems that higher-molecular-weight hyaluronic acid may be more effective than lower molecular-weight HA. Intra-articular treatment with HA has been accepted and is widely used as OA therapy. However, there is a controversy over the efficacy of orally administered HA. 

Based on basic pharmacokinetic research it has been found that orally administered high-molecular-weight HA also reached the joint [83], which provides a rationale for the oral supplementation of HA. Authors of a clinical pilot study [84] concluded that HA enhances several aspects of quality of life in adults with knee OA. A larger sample size would be necessary to confirm this result.

In a recent review in which the SYSADOA treatment was analyzed using the GRADE system [33], experts came to the conclusion that—in addition to chondroitin sulfate or glucosamine sulfate—also hyaluronic acid has “demonstrated pain reduction and physical function improvement with very low toxicity, with moderate to high quality evidence” [33]. In summary, the described effects justify the use of these three cartilage components in patients suffering from OA.

For *collagen hydrolysate*, from the available *in vitro* and* in vivo* studies as well as clinical trials [85, 86], it may be concluded that collagen hydrolysate is absorbed by the gastrointestinal tract and incorporated into the joint cartilage. It may lead to increased mobility and physical function with a significant pain relief. 

## 4. Anti-Inflammatory and Antioxidant Effects of Nutrients

### 4.1. Inflammation and Reactive Oxygen Species: New Metabolic Approaches to Osteoarthritis

**Figure 2:**
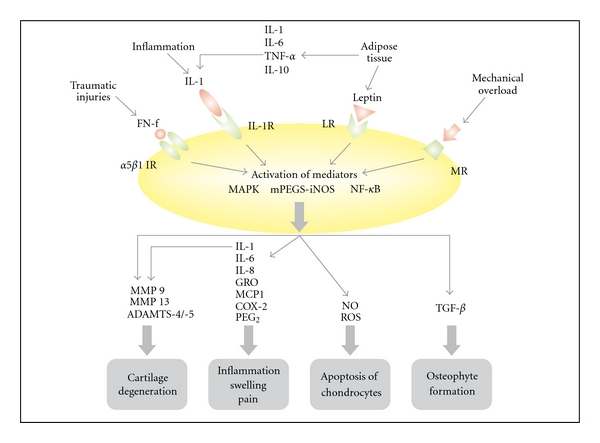
Inflammatory and oxidative processes involved in OA; FN-f: fibronectin fragment; IL-1 R: interleukin receptor; IR: integrin receptor; LR: leptin receptor; MR: mechanoreceptor.

While OA is not synonymous with inflammatory arthropathy, new results indicate that inflammation is not only a secondary event, it is involved in the development of OA from the very beginning [87–89]. Many inflammatory mediators are expressed in the cartilage and synovial tissue in early OA stages. The findings of Benito [89] indicate that inflammatory mediators and nuclear transcription factors involved in the inflammatory cascade are significantly higher in early-stage OA patients, when compared to late-stage OA. Additionally, reactive oxygen species (ROS) increase during OA [90–93]. The various inflammatory and oxidative processes in OA are summarized in Figure 2.

Many studies have identified *overweight* (BMI 25–29.9 kg/m^2^) and *obesity* (BMI >29.9 kg/m^2^) [94–96] as major OA risk factors. Hart and Spector [97] showed that a BMI increase of 2 units will increase the risk of knee OA manifestation by 36%. This is not only due to the additional weight and mechanical stress on the joints, as nonweight-bearing joints—such as the hands—are significantly more affected in patients with high BMI [89], due to metabolic reactions. These include increased inflammation, induced by leptin and other adipocytokines, and dietary lipids or lipid peroxidation, which can lead to cartilage destruction. Therefore, OA is not induced by biomechanical factors and age alone, and several metabolic factors are also involved [98–106].

*Leptin is overexpressed in obese patients* and is present in the synovial fluid, as well as articular chondrocytes [104]. Chondrocytes in joint cartilage also express leptin receptors [107]. Under physiological conditions, leptin stimulates the synthesis of insulin-like growth factor 1 (IGF-1) and transforming growth factor beta (TGFb-1), two mediators important for proliferation of chondrocyte and extracellular matrix synthesis, by binding to the leptin receptor [103, 104]. These two factors appear to have a positive anabolic impact on the joint by increasing the cartilage matrix production. Excessive and pathological concentrations of leptin, however, like those found in obese patients, have an opposite effect on chondrocytes, cartilage, and bone, leading to osteophyte formation and cartilage degeneration [108]. Osteophytes in the joints usually limit joint movement and thus provoke pain.

*In vitro* experiments have elucidated several mechanisms by which excessive amounts of adipokines lead to the destruction of articular joints. In cartilage derived from human OA patients, leptin enhances the synthesis of several proinflammatory mediators, such as NO, PGE_2_, IL-6, and IL-8, via inducible nitric oxide synthase (iNOS) pathways. By inhibiting the iNOS activity, NO synthesis was nearly completely blocked. This reduction of NO reduces the production of PGE_2_, IL-6, and IL-8 [109]. Furthermore, membrane bound prostaglandin E synthase 1 (mPGES-1) and COX-2 enzyme are overexpressed in the cartilage of such patients. COX-2 further increases the production of prostaglandins. This overexpression can be induced by IL-1 and TNF-alpha, factors released by adipose tissue. mPGES-1 mediates the production of PGE_2_ [110]. PGE_2_ overproduction enhances NO-induced cell death of OA chondrocytes [111]. When IL-1 acts together with leptin, they can activate nitric oxide synthase type II, which increases NO production in chondrocytes [112]. Elevated NO levels lead to various catabolic processes in the cartilage, such as the loss of chondrocyte phenotype, thereby reducing production of ECM, and to chondrocyte apoptosis, and ECM degradation [113, 114].

Leptin induces the synthesis of matrix metalloproteinases (MMP), especially MMP9 and MMP13 [115–117], via IL-1 and TNF-alpha. MMPs are a large family of enzymes that degrade different components of collagen and proteoglycans [118]. Both MMP9 (gelatinase) and MMP13 (collagenase) are involved in cartilage damage [116, 117]. MMP13 is produced by chondrocytes and cleaves collagen type II (the main collagen type in articular cartilage) and the proteoglycan molecule aggrecan, leading to structural damage of the cartilage tissue [115]. These experiments clearly show that obesity, mediated by leptin, exerts a proinflammatory and catabolic effect on cartilage, leading to apoptosis of chondrocytes and the degradation of the extracellular matrix.

Leptin is not the only adipokine associated with inflammatory actions. Resitin and visfatin, together with leptin, increase the inflammatory status by means of various mechanisms, which together with mechanical overload leads to phenotype loss and apoptosis of chondrocytes, as well as cartilage matrix degeneration [99, 101].

Thus, overweight and obesity play an important role in the genesis of knee and hip joint OA not only as a result of mechanical overload but also by the complex combined action of genetic, metabolic, neuroendocrine, and biomechanical factors and represent a *significant modifiable risk factor* [102] not least for this reason.

Inflammation is also induced by *overloading *the joints. Various mechanoreceptors are expressed on the surface of chondrocytes. It has been reported that mechanical compression significantly increases PGE_2_ release in chondrocyte explants. It was shown that mechanical stress induced COX-2 expression and that mPGES-1 mRNA (PGE synthase 1) and protein are increased in cartilage explants. mPGES-1 is involved in PGE_2_ synthesis during inflammation. PGE_2_ is most likely a key regulator of cartilage degeneration in OA [119]. mPGES-1 and COX-2 have also been found to be stimulated by IL-1 in chondrocytes [110].

*Traumatic injury* to the joints results in activation of many genes, including inflammatory mediators, cartilage degrading proteinases, and stress response factors [3]. Degeneration of the cartilage leads to fibronectin fragments (FN-f). Fibronectin and fibronectin fragments are found in the synovial fluid after traumatic injuries. Investigators were able to show that these fragments stimulate the expression of inflammatory cytokines and chemokines, such as IL-8, IL6, and IL-1, indicating that cartilage damage can result in further progressive cartilage degradation. The stimulation of the cytokines by FN-f is mediated by the NF-*κ*B pathway [120]. It was further shown that FN-f stimulates MMPs in chondrocytes, which breaks down the cartilage [121, 122]. MMP13, for example, destroys type II collagen, the main collagen component of the hyaline cartilage [123, 124].

Regardless of the source, increased concentrations of inflammatory mediators activate specific aggrecanases (ADAMTS-4/-5), which cleave the aggrecan molecule in a specific region and thereby destroy the activity of this important cartilage structure molecule [125]. 

Inflammation and oxidative stress are prominent mechanisms which lead to progression of OA. Thus, therapy must also address this aspect.

### 4.2. How Can Nutrients Modulate Inflammation Processes and Oxidative Stress Involved in Osteoarthritis?

The complex relationship between obesity and OA shows that overweight certainly represents the most significant modifiable risk factor for avoiding knee or hip joint OA. Weight reduction and weight stabilization on the basis of a balanced diet with low energy density is crucial in manifest OA [127]. But also the metabolic processes can be influenced by a dietary therapy which mainly includes chondroprotectives, such as glucosamine and chondroitin sulfate or omega-3 fatty acids. 

An alternative treatment to the common NSAID therapy for OA is the use of so-called nutraceuticals, such as glucosamine, chondroitin sulfate, hyaluronic acid, hydrolyzed collagen, and omega-3 fatty acids and various vitamins and minerals. In addition to cartilage metabolism stimulation and thereby cartilage regeneration, many of them possess mechanisms which modulate the inflammatory events and oxidative processes involved in OA. As they are components of natural foods, they have far fewer adverse effects in long-term use than NSAIDs or COX-2 inhibitors, as shown in many clinical trials (see above). 

They interfere with the inflammatory scenario, illustrated above, at various points (see also Figure 2).

The glucosamine and chondroitin sulfate combination suppresses IL-1-induced gene expression of iNOS, COX-2, mPGEs, and NF-*κ*B in cartilage explants. This leads to reduced production of NO and PGE_2_, two mediators responsible for the cell death of chondrocytes and inflammatory reactions [128, 129]. There are several ways by which glucosamine or chondroitin sulfate reduce synthesis of the COX-2 enzyme. Inhibition of the IL-1 beta induced NF-*κ*B pathway by glucosamine sulfate results in reduced synthesis of the COX-2 enzyme [130–133]. Another manner in which glucosamine hydrochloride inhibits COX-2 activity is the prevention of COX-2 co-translational N-glycosylation and the facilitation of COX-2 protein turnover [134]. CS alone diminishes the nuclear translocation of NF-*κ*B, which reduces the formation of proinflammatory cytokines IL-1beta and TNF-alpha and proinflammatory enzymes such as cyclooxygenase 2 (COX-2) and nitric oxide synthase-2 (NOS-2) (for review see [135]).

The anti-inflammatory capability of CS was also tested in a rabbit atherosclerosis model. In that model, CS reduced the proinflammatory molecules C-reactive protein and IL-6 in serum, as well as the expression of MCP-1 and COX-2 in the peripheral blood mononuclear cells. It also influenced NF-*κ*B [136] that is responsible for the induction of inflammatory processes. 

Additionally, inflammation mediators activate various cartilage degenerating enzymes. The mRNA expression of such enzymes (MMP-13 and aggrecanases (ADAMTS-5)) was reduced in cartilage explants incubated with GlcN·S and CS. In the same study, the tissue inhibitor of metalloproteinase-3 (TIMP-3), a potent inhibitor of ADAMTS, was upregulated [128]. Glucosamine sulfate alone was shown to inhibit the activation process of MMP-2 and MMP-9 expression, via downregulation of the NF-*κ*B pathway [137].

Inflammatory mediators are responsible for reduced biosynthesis of cartilage material. Experiments with rat chondrocytes have shown that IL-1*β* inhibits the expression of the enzyme galactose-*β*-1,3-glucuronosyltransferase I (GlcAT-I), a key enzyme in the biosynthesis of cartilage GAG chains. Dose-dependently glucosamine was able to reduce this inhibition [132].

In addition to their anti-inflammatory action, glucosamine and chondroitin sulfate exhibit an antioxidant action which leads to a significant reduction in iNOS expression and activity [138, 139]. This is one explanation why glucosamine and chondroitin reduce the otherwise NO-induced cell death of chondrocytes. In comparison to glucosamine and CS, hyaluronic acid exerted a very minor anti-inflammatory and antiapoptotic effect, while it significantly reduced NO levels [139].

**Vitamins and Minerals**

Many vitamins are known for their antioxidant capacity. Under physiological conditions, the reactive oxygen species (ROS), produced by the body are neutralized by the body's antioxidant defense system, such as peroxidase, superoxide dismutase, or catalase. Under disease conditions, however, the increased amount of ROS can no longer be managed by the natural defense system. Arthropathies such as osteoarthritis and rheumatoid arthritis are characterized by the increased formation of free radicals [98, 102, 140]. ROS, which are extensively expressed during OA [92, 93, 141, 142], are involved in matrix and cartilage degeneration, inhibition of matrix synthesis, cell death, and apoptosis of chondrocytes. *In vitro* experiments confirmed that mechanical shear stress increases the production of oxidants in cartilage explants [90].

In a study, serum samples of 29 patients with knee OA and 26 healthy controls were analyzed for their oxidative status [92]. Total antioxidant capacity (TAC) and, in addition, the oxidative stress index (OSI Index) were determined as antioxidant parameters. The oxidative stress was measured based on total peroxide (TP) content and lipid hydroperoxide and the OSI Index was calculated from the TP/TAC ratio. Compared with the healthy controls, the OA patients had a significantly higher OSI Index, whereas all the markers for antioxidant activity were lower. Prolidase activity (collagen synthesis marker) was also significantly lower in the OA patients. Moreover, the enzyme activity correlated positively with the antioxidant concentration (TAC) and negatively with oxidative stress (OS). Hence, the higher the antioxidant concentration, the better the cartilage metabolism process. Conversely, oxidative stress was associated with impaired cartilage metabolism [92].

A working group showed that OA patients have a significantly reduced concentration of antioxidants (vitamins C and E) and increased oxidative stress. Oxidative stress was measured on the basis of the malondialdehyde (MDA) concentration [93].

Therefore, OA treatment should not only focus on regeneration and anti-inflammatory processes but also on the reduction of oxidative stress in these patients. Positive effects on OA have been found for a number of vitamins and minerals (for review see [143, 144]).

*Vitamin C*, for example, stimulates collagen synthesis, and to a lesser extent the synthesis of aggrecan. Proteoglycan synthesis is increased in chondrocyte cultures [145] (for review see [143, 146]). An animal study showed that vitamin C has a protective effect on knee cartilage [147]. The effect of chondrocyte protection could be mediated by its antioxidant capacity. Similar results were reported for vitamin E, which is known for its strong antioxidant effects, its protection against ROS, and enhancement of chondrocyte growth [143]. The positive effects of vitamin E were demonstrated in clinical trials. Patients treated with vitamin E displayed a significant reduction in pain when compared to placebo, and comparable effects to diclofenac (for review see [143]).

*Selenium, zinc, and copper* are minerals under discussion as supporting OA treatment. They exhibit antioxidant characteristics and are part of antioxidant enzymes. Rats fed with a low selenium diet showed a decrease in sulfotransferase activity. This enzyme is involved in the process of glycosaminoglycan synthesis, which is important for the cartilage matrix [148]. In a double-blind, placebo-controlled study, the combination of *selenium and vitamins A, C, and E,* had a positive but nonsignificant effect that tends to improve pain and stiffness in OA patients, compared to placebo [149]. *Manganese* is a component of glycosal and xylosyltransferase enzyme which are responsible for the glycosidic binding and thus for the glycosaminoglycan synthesis. Manganese is also involved in the cross-linking of collagen fibrils and inhibits elastin-degrading elastases [150]. Copper, an essential component of lysyl oxidase, contributes to the cross-linking of collagen and elastin in cartilage and bone tissue, and molybdenum is a cofactor of sulfitoxidase enzyme producing sulfates which are important for proteoglycan synthesis.

**Synergistic Action of Chondroprotectives, Omega-3 Fatty Acids, and Other Nutrients**

*Omega-3 polyunsaturated fatty acids* (PUFAs), such as linolenic acid and eicosapentaenoic acid (EPA), are found in walnut, flaxseed, and fish oils. They are known for their anti-inflammatory actions, which has been shown in several studies (see [144, 147, 151]). They have been successfully used in clinical trials, mainly to treat rheumatoid arthritis [152–154]. *In vitro* studies showed that omega-3 fatty acids increase collagen synthesis and decrease the inflammation mediator PGE_2_ [155]. EPA, when oxygenated, results in the bioactive product resolving E1 (RvE1). By activation of a specific receptor, ChemR23, RvE1 dramatically reduces inflammatory processes by inhibiting the NF-*κ*B pathway that is responsible for many of these processes [156]. Omega-3 fatty acids decrease IL-1-induced aggrecanase and collagenase activity and reduce mRNA expression of ADAMTS-4, COX-2, IL-1*α*, and TNF-*α*. Furthermore, they decrease the protein levels of several MMPs [157] (for review see [144]).

*PUFAs* are important components of a dietary OA therapy. Oxygen radicals are eliminated through the supplementation of antioxidants. They are generated to an increased extent in OA and are involved in cartilage degeneration (99,152), but also promote inflammatory processes in the body quite generally. Numerous studies have dealt with the anti-inflammatory effects of the polyunsaturated fatty acids *eicosapentaenoic acid (EPA) and docosahexaenoic acid (DHA)* and their role in cartilage metabolism [157].

**Figure 3:**
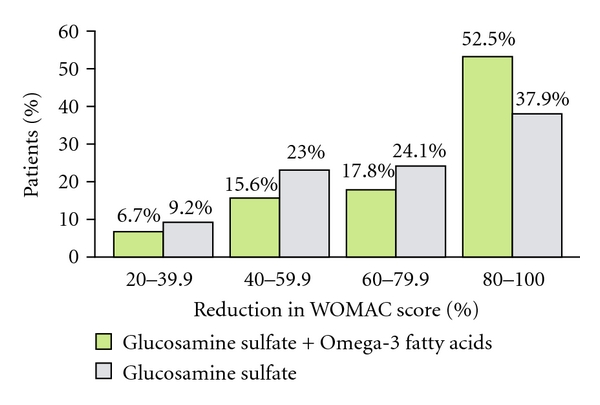
A greater proportion of patients with combination therapy GlcN·S + O-3 FA showed the highest WOMAC improvements of 80–100% [126].

A recent study was able to demonstrate that the combined administration of EPA and DHA in a glucosamine therapy markedly alleviated the discomfort of knee and hip joint OA patients [126]. In this randomized study, 177 patients suffering from moderate to severe OA of the knee or hip joint were subdivided in two groups. One group took a combination of 1,500 mg of glucosamine sulfate plus the omega-3 fatty acids EPA and DHA as well as vitamins A, D, and E every day for 26 weeks. The other group was given a preparation without EPA and DHA. At baseline and at weeks 13 and 26 the subjects were examined and their complaints were documented based on the Western Ontario and McMaster Universities Osteoarthritis-Index (WOMAC). Both groups showed an improvement as a result of the therapy demonstrated by a reduction in the WOMAC pain score of 20% or more. If the criterion of therapy success was greater, for example, 80%, a significantly greater number of patients in the combination group (52.2%) reached this aim as compared to the group taking the preparation without EPA and DHA (37.9%; *P* = 0.044; Figure 3). In addition, typical OA symptoms such as joint stiffness or joint pain had already decreased at week 13 and towards the end of the trial continued to decrease by 48.5% to 55.5% in the EPA and DHA group as compared to 41.7% to 55.3% in the control group.

The results of these *in vivo* and *in vitro* experiments clearly demonstrate an anti-inflammatory action for glucosamine, chondroitin sulfate, hyaluronic acid, and omega-3 fatty acids. Due to these abilities, it is plausible that such nutrients can reduce collagen degradation [157] in osteoarthritis.

## 5. Conclusion

Based on the preclinical and clinical data, it is obvious that chondroprotectives such as glucosamine, chondroitin sulfate, and other nutrients, such as antioxidants and PUFAs, can modulate osteoarthritis. In long-term use they exhibit, in contrast to NSAIDs, an excellent safety profile, with as few adverse events as placebo.

The chondroprotectives are essential components of the cartilage metabolism and stimulate important cartilage regeneration processes, thereby adjusting the imbalance of catabolic and anabolic processes in osteoarthritis.

Newer data point out that inflammation and oxidative stress are characteristics of all stages of the disease. Chondroprotectives are able to inhibit many of these processes. They defend chondrocytes against oxidative stress-induced apoptosis, reduce the inflammatory mediator-induced joint cartilage degeneration, and reactivate the inflammation-reduced anabolic processes of extracellular matrix components. This leads to reduced inflammation, swelling, and pain, and to an increased mobility of the affected joints. Especially when used in combination with other nutrients, such as antioxidants and omega-3 fatty acids, these substances are able to exert synergistic effects on the osteoarthritic joints.

Recently new study results were published that demonstrate promising effects of further food substances or phytochemicals, such as contained in ginger extracts, showing various antiosteoarthritic actions and, for example, even intra-articular resveratrol showing chondroprotective effects in a rat animal model.

In summary, future “nutraceutical” approaches to OA most likely will have to be more complex and should include glucosamine sulfate (and/or chondroitin sulfate) together with hyaluronic acid, collagen hydrolysate, and several other nutrients which were shown to have promising actions on joint cartilage, synovial fluid, and overall clinical outcome in OA patients.

## Figures and Tables

**Table 1:** Characteristics and results of selected placebo-controlled trials.

Author(s), year	Agent/Doses	Duration	Pts (*n*)	Outcome measure	Results and conclusion
Bruyere et al. 2008 (follow-up of two RCTs 2001/02-see below) [27]	GlcN·S/1500 mg	Formerly: 3 yrs Now: mean 5 yrs after trial termination	340∗(275 = 81% retrieved) GlcN·S: 144 Plac: 131∗ at least 12-month treatment	Incidence of total knee replacement	*Formerly*: knee OA progression reduced, structure- and symptom-modifying effects *Now*: 6.3% of GlcN·S pts underwent total knee replacement surgery versus 14.5% of the plac. pts *P* = 0.026 Risk of total knee replacement could be reduced 5 yrs after drug discontinuation.
Clegg et al. 2006 (GAIT, Glucosamine/Chondroitin Arthritis Intervention Trial) [28]	GlcN·HCl/1500 mg CS/1200 mg Celecoxib 200 mg	6 months	1583	20% reduction of knee pain	GlcN·HCl + CS: 66.4% of pts. had 20% pain reduction versus Plac: 61.1% of pts. *P* = 0.09—n.s.
			354 (subgroup with moderate-to-severe pain)	20% reduction of knee pain	GlcN·HCl + CS: 79,2% versus Plac. 54.3% *P* = 0.002 Combination of GlcN·HCl + CS was effective in reducing moderate-to-severe pain in knee OA.
Herrero-Beaumont et al. 2007 (GUIDE, Glucosamine Unum In Die Efficacy) [29]	GlcN·S/1500 mg Acetaminophen 3000 mg	6 months	318 GlcN·S 106 Acet. 108	OARSI-A responder (relative change WOMAC pain subscale of at least 55%)	GlcN·S: 39.6% versus plac. 21.2% *P* = 0.004 Acet.: n.s. GlcN·S was superior to plac. in the treatment of knee OA
Reginster et al. 2001 [30]	GlcN·S/1500 mg	3 yrs	212: GlcN·S 106	Radiographs of the knee: joint spacenarrowing; Lequ. index, WOMAC score	GlcN·S: no significant joint space loss, WOMAC score reduction Plac.: progressive joint space narrowing, WOMAC score increased: Structure- and symptom-modifying effects
Pavelka et al. 2002 [31]	GlcN·S/1500 mg	3 yrs	202: GlcN·S 101	Radiographs of the knee: joint space narrowing; Lequ. index, WOMAC score	GlcN·S: no significant joint space loss Plac.: progressive joint space narrowing *P* = 0.001 GlcN·S: Lequ. and WOMAC scores improved by 20% to 25%; Structure- and symptom-modifying effects
Bruyere et al. 2004 [32]	GlcN·S 1500 mg	3 yrs	319 postmenopausal women (of 414 pts of two RCTs, see above)	Radiographs of the knee: joint space narrowing; WOMAC score	GlcN·S: no significant joint space loss Plac.: progressive joint space narrowin *P* < 0.0001 WOMAC score reduction: “Pain” (*P* < 0.02) and “Function” (*P* < 0.004)

**Table 2:** Characteristics and results of selected reviews/meta-analyses-glucosamine.

Author(s), year	Analyzed publications	Trial details	Conclusions
Bruyere et al. 2008 [33]	(i) Towheed et al., Cochrane Review 2005 [34]	20 RCTs: GlcN·S superior to Plac. with a 28% improvement in pain and a 21% improvement in function (Lequ. index).	Significantly superior to placebo in terms of its ability to reduce levels of pain.
	(ii) Vlad et al. 2007 [35]	15 RCTs Summary effect sizes ranged: 0.05 to 0.16 in trials without industry involvement, but 0.47 to 0.55 in trials with industry involvement.	Heterogeneity among trials of glucosamine is larger than would be expected by chance. Glucosamine hydrochloride is not effective.
	(iii) Reginster 2007 [36] (update following Richy et al. 2007, [37])	3 pivotal RCTs: WOMAC pain and function subscores: significant beneficial effect of GlcN·S versus Plac.	The effect size was consistent across the parameters, and it was approx. 0.30 or slightly higher. This effect is small to medium, but it is clinically valid (>0.20), and especially, it is of the same magnitude as that commonly encountered with other OA treatments, including NSAIDs.

Poolsup et al. 2005 [38]		14 RCTs: GlcN·S: Risk of disease progression was reduced by 54% (*P* = 0.0011). Pooled effect sizes for pain reduction and improvement in physical function were 0.41 (*P* < 0.0001) and 0.46 (*P* < 0.0001), respectively.	GlcN·S may be effective and safe in delaying the progression and improving the symptoms of knee OA.

**Table:** (a)

Author(s), year	Analyzed publications	Trial details	Conclusions
Uebelhart 2008 [39]	Meta-analysis	3 RCTs with CS in knee OA: 462 pts., 2 × 3 mo. 800 mg for 1 yr; 800 mg daily and continuously for 12 and 24 months. 2 RCTs with CS in finger joint OA: 284 pts., 3 × 400 mg CS for 3 yrs. CS decreased the number of pts. with new erosive OA finger joints.	CS influences the symptoms of OA such as pain and inflammation, but also acts as a structure-modifying drug in OA (SMOAD). CS may retard OA progression and could modify the course of OA.

Lee et al. 2010 [40]	Meta-analysis	2 RCTs with GlcN·S + 4 RCTs with CS (800 mg daily) in OA: 1502 pts. CS: Small, but significant protective effect on minimum joint space narrowing after 2 years (*P* < 0.001).	CS may delay radiological progression of OA of the knee after daily administration for over 2 years.

Hochberg et al. 2008 [41]	Meta-analysis	3 RCTs with CS in knee OA: Small significant effect on the reduction in rate of decline in minimum joint space width of 0.07 mm/year. The effect size is 0.26 (*P* < 0.0001).	CS is effective for reducing the rate of decline in minimum joint space width in OA of the knee; CS may have a role as a structure-modifying agent in the management of patients with knee OA.

**Table:** (b)

Author(s), year	CS/Dose	Duration	Pts. (*n*)	Outcome measure	Results and conclusion
Kahan et al. 2009 (STOPP: Study on Osteoarthritis Progression Prevention) [42]	CS/800 mg	2 yrs	622 (knee OA) CS: 309	X-ray images, tibiofemoral joint: joint space narrowing	Progression of joint space narrowing was significantly reduced versus plac. (28% CS pts. versus 41% Plac. pts. showed progressive joint space narrowing, *P* < 0.0005) Combined structure- and symptom-modifying effects of CS suggest that it could be a disease-modifying agent in patients with knee OA.

Michel et al. 2005 [43]	CS/800 mg	2 yrs	300 (knee OA) CS: 150	X-ray images, tibiofemoral joint: joint space narrowing	CS: no significant joint space loss, *P* = 0.04 versus Plac. Plac.: significant joint space narrowing (*P* = 0.001 versus baseline) CS: no significant symptomatic effect, but halts structural changes in OA for over 2 yrs.

## Abbreviations

CS: Chondroitin sulfate
COX: Cyclooxygenase
ECM: Extracellular matrix
EULAR: The European League Against Rheumatism
FN-f: Fibronectin fragment
GAG: Glycosaminoglycan
GlcN: Glucosamine
GlcN·HCl: Glucosamine hydrochloride
GlcN·S: Glucosamine sulfate
IL: Interleukin
IR: Integrin receptor
JSW: Joint space width
LR: Leptin receptor
MMP: Matrix metalloproteinase
MR: Mechanoreceptor
NF-*κ*B: Nuclear factor-*κ*B
iNOS: Inducible nitric oxide synthase
NSAIDs: Nonsteroidal anti-inflammatory drugs
OA: Osteoarthritis
PGE_2_: Prostaglandin E_2_
ROS: Reactive oxygen species
SMOAD: Structure-modifying OA drug
SYSADOA: Symptomatic slow-acting drug in osteoarthritis
WOMAC: Western Ontario and McMaster Universities.
